# Hybrid Suture- and Plug-Based Closure Versus Dual Suture Devices in Transfemoral Transcatheter Aortic Valve Replacement: A Systematic Review and Meta-analysis

**DOI:** 10.1016/j.jscai.2026.105339

**Published:** 2026-05-14

**Authors:** Abdul Moeez, Syed Wajihullah Shah, Sundus Huma, Umm E. Salma Shabbar Banatwala, Aizaz Ali, Lintha Zafar Khattak, Zaryab Bacha, Afra Khan, Fazlina Shaid, Touba Azeem, Sayyam Razzaq, Malik Waleed Zeb Khan, Jibran Ikram, Farid Ullah, Mohammed Shaban

**Affiliations:** a Department of Medicine, Lady Reading Hospital, Peshawar, Pakistan; b Department of Medicine, Khyber Medical College, Peshawar, Pakistan; c Department of Medicine, Dow University of Health Sciences, Karachi, Pakistan; d Department of Medicine, Khyber Teaching Hospital, Peshawar, Pakistan; e Department of Medicine, Poonch Medical College, Rawalkot, Pakistan; f Department of Internal Medicine, AdventHealth Orlando, Orlando, Florida; g Cardiovascular Medicine, Department, Heart, Vascular & Thoracic Institute, Cleveland Clinic, Cleveland, Ohio

**Keywords:** aortic valve stenosis, postoperative hemorrhage, transcatheter aortic valve replacement, vascular closure devices

## Abstract

**Background:**

Effective vascular closure is crucial in transfemoral transcatheter aortic valve replacement (TAVR) to minimize access-related complications. Although dual suture-based closure is widely used, a hybrid approach combining suture- and plug-based vascular closure devices (VCD) has gained increasing attention. This meta-analysis compared these 2 strategies in terms of vascular outcomes and procedural success.

**Methods:**

A systematic review and meta-analysis were conducted according to the Preferred Reporting Items for Systematic Reviews and Meta-Analysis (PRISMA) guidelines. PubMed, Embase, and Web of Science were searched through January 2025 for studies comparing suture-plus-plug–based vs suture-based VCD in TAVR patients. Primary outcomes were major and minor vascular complications, bleeding events, VCD failure, and unplanned interventions. Risk ratios (RRs) with 95% CIs were calculated.

**Results:**

Six studies involving 2308 patients were included. The suture-plus-plug–based approach significantly reduced major vascular complications (RR, 0.50; *P* = .001), minor vascular complications (RR, 0.58; *P* < .00001), and VCD failure (RR, 0.26; *P* < .00001) compared with suture-based closure alone. No significant differences were observed in major (*P* = .30) or minor bleeding (*P* = .47). Unplanned interventions were less frequent with the hybrid approach, but the difference was not statistically significant (RR, 0.68; *P* = .19).

**Conclusions:**

The combination of suture- and plug-based closure devices in transfemoral TAVR provides superior vascular outcomes and lower device failure rates without increasing bleeding risk. These findings support the hybrid approach as a safe and effective strategy, although further randomized controlled trials are warranted to confirm long-term outcomes.

## Introduction

Transcatheter aortic valve replacement (TAVR) has become an established, less invasive alternative to surgical aortic valve replacement for severe aortic stenosis across all surgical risk profiles, with the transfemoral (TF) approach emerging as the predominant access route, exceeding 97% implementation in many high-volume centers.^1^^,^^2^ However, despite significant technological advances in valve design and delivery systems, the necessity for large-bore arterial access (typically 14F-20F) in patients with advanced age and multiple comorbidities, including peripheral arterial disease, creates a substantial risk for access-site complications, as vascular complications remain prevalent in 5% to 17% of cases despite advancements in device profiles and procedural techniques.^3^^,^^4^ These complications—including access-site hematomas, pseudoaneurysms, retroperitoneal bleeding, and arterial dissection—correlate with prolonged hospitalization, increased transfusion requirements, and elevated 30-day mortality.^5^ Vascular closure devices (VCD) play a crucial role in achieving hemostasis following large-bore arterial access during TAVR procedures. Currently, 2 major categories of VCD are utilized in clinical practice: suture-based devices (SBD) and plug-based devices (PBD).^1^^,^^6^^,^^7^ Recent evidence suggests that plug-based systems may offer certain advantages in specific patient populations, particularly regarding time to hemostasis and bleeding complications.^8^ However, randomized trials revealed contradictory findings: the MASH trial reported comparable vascular complications between plug-based closure and dual suture closure but higher modified VCD failure with the suture-based technique,^9^ whereas the CHOICE-CLOSURE trial demonstrated significantly higher access-site complications with pure plug-based strategies.^7^ This divergence underscores the limitations of relying on single-device strategies.

A paradigm shift emerged with the concept of combined VCD utilization, integrating suture- and plug-based mechanisms. Mechanistically, this hybrid approach addresses the weaknesses of individual devices: SBD mitigate anchor-related bleeding risks inherent to PBD, whereas PBD provide immediate hemostasis in cases of suture failure.^10^ Despite these advances, critical evidence gaps persist. The evidence supporting this dual-device approach remains fragmented and inconsistent across different clinical settings. Various factors, including patient characteristics, procedural aspects, operator experience, and center-specific protocols, may influence the outcomes of combined closure strategies. Current syntheses overlooked combined strategies, which constituted <5% of analyzed cases.^11^ Furthermore, heterogeneity in Valve Academic Research Consortium (VARC)-2 adherence, operator experience, and calcification severity (a modifier of PBD efficacy per CHOICE-CLOSURE subgroup analyses) complicates cross-study comparisons.^12^^,^^13^ Additionally, the economic implications and resource utilization associated with employing multiple VCD need to be carefully balanced against the potential clinical benefits. Therefore, we conducted this systematic review and meta-analysis to comprehensively evaluate the efficacy and safety of the combined use of SBD and PBD for femoral access hemostasis in TAVR procedures compared to traditional single-device approaches. By synthesizing the available evidence, we aim to provide insights that can guide clinical decision-making and inform future research directions in this rapidly evolving field.

## Methods

### Study design and protocol registration

The meta-analysis was completed according to the guidelines set forth in the Preferred Reporting Items for Systematic Reviews and Meta-Analysis (PRISMA) statement,^14^ as well as the protocols outlined in the Cochrane Handbook for systematic reviews and meta-analyses.^15^ Our protocol has been registered in PROSPERO with a designated registration ID: CRD42025644707.^16^

### Search strategy and databases

A comprehensive search of the PubMed, Web of Science, and Embase databases was undertaken, covering all the available entries from their inception until January 2025. We conducted a search in English without language restrictions or search limits, using the following keywords: “Aortic Valve Stenosis,” “Transcatheter Aortic Valve Replacement,” “Vascular Closure Devices,” and “Postoperative Hemorrhage.” The detailed search string and the results are outlined in Supplemental Table S1. A manual search of the reference sections of the included studies was conducted to identify additional relevant studies.

### Eligibility criteria

The criteria for included studies were defined as follows: population: “Patients undergoing TAVR”; intervention: “Combination of suture-based closure device with a plug-based closure device”; control: “Dual suture based closure”; outcomes: “Major and minor vascular bleeding, major and minor vascular complications, VCD failure”; study type: “high-quality cohort studies and randomized controlled trials (RCTs).”

Studies involving patients undergoing procedures other than TAVR or combined with other surgeries not focused on TAVR, studies that do not specifically assess the impact of SBD in combination with a PBD vs dual suture or dual SBD, case reports, review articles, meta-analyses, conference abstracts, editorials, animal-based studies, and duplicate studies were excluded.

### Study selection

**Figure 1:**
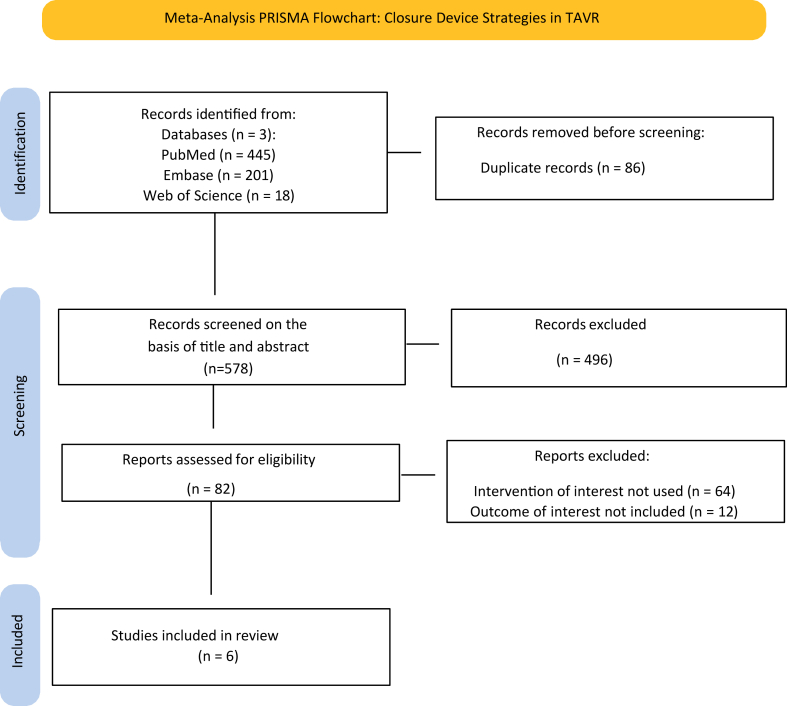
**Preferred Reporting Items for Systematic Reviews and Meta-Analysis (PRISMA) flowchart for study selection in the meta-analysis of closure device strategies in transcatheter aortic valve replacement (TAVR)**.

The articles from all 3 databases were imported to Rayyan software (Rayyan) for screening. Duplicate studies were removed automatically by the software. Two authors (S.H. and A.K.) independently reviewed the articles based on title and abstract. Subsequently, a comprehensive review of the full text of selected articles was conducted, based on our established inclusion criteria. Any discrepancies were addressed by the lead author. The details of the selection process are illustrated in the PRISMA flowchart (Figure 1).

### Data extraction

We conducted a preliminary extraction after obtaining the full texts from the relevant studies. This was done to create an extraction template in Excel (Microsoft). This was in 2 sections: in the baseline: first author's name, year of publication, participants, intervention/control, age, body mass index, sheath-to-femoral artery ratio, atrial fibrillation, hemoglobin, previous myocardial infarction, and hypertension.

The primary and secondary outcomes included major and minor vascular complications, major and minor vascular bleeding, VCD failure, and unplanned surgical site interventions. Major and minor vascular complications and bleeding were defined according to standardized criteria commonly used in TAVR research, incorporating parameters such as access-site hematoma, pseudoaneurysm, arterial dissection, retroperitoneal hemorrhage, hemoglobin drop, and transfusion requirements. VCD failure was identified as incomplete hemostasis requiring additional interventions, while unplanned interventions referred to any unexpected procedures performed to manage vascular complications. To ensure consistency in bleeding outcome assessment, the analysis for major and minor vascular bleeding was based on studies that applied uniform bleeding definitions (Appendix). For vascular complications, all included studies used comparable criteria, allowing pooled analysis across the entire dataset (Appendix). Three authors (Z.B., L.Z.K., and S.W.S.) independently extracted data from the selected studies using a standardized template in Excel. Any discrepancies were resolved by the first author.

### Quality assessment and risk of bias

To assess the risk of bias in our study, we employed the revised Cochrane Collaboration tool for RCT (RoB 2)^17^ for all the randomized controlled trials. The evaluation indicators included randomization sequence generation, allocation concealment, blinding, incomplete outcome data, and selective reporting. The Newcastle-Ottawa Scale was used to evaluate the quality of nonrandomized studies based on selection, comparability, and exposure. Both tools classified the risk of bias in the included studies as low, unclear, or high.

### Statistical analysis

Statistical analysis was performed using RevMan version 5.4 (The Cochrane Collaboration). A fixed-effects model was used unless the pooled studies showed considerable heterogeneity (I^2^ > 50%), at which point the random-effects model was used instead.^18^ All the dichotomous outcomes were reported as risk ratios (RRs) with a 95% CI. We employed the χ^2^ test and the I^2^ statistic to evaluate and quantify the level of heterogeneity in the data. A *P* value <.10 from the χ^2^ test indicated significant heterogeneity. For the I^2^ statistic, a range from 30% to 60% signifies moderate heterogeneity, whereas a value exceeding 60% indicates significant heterogeneity. When I^2^ was >30%, a sensitivity test was conducted to address it. An overall *P* value <.05 was regarded as statistically significant.^19^

## Results

### Search results

A comprehensive literature search was conducted using 3 electronic databases: PubMed (n = 445), Embase (n = 201), and Web of Science (n = 18), yielding a total of 664 records. After the removal of 86 duplicate records, 578 unique records were screened based on title and abstract. Following the initial screening, 496 records were excluded.

A total of 82 full-text reports were assessed for eligibility. Of these, 64 studies were excluded as they did not utilize the intervention of interest, and 12 studies were excluded because of the absence of relevant outcome data. Ultimately, 6 studies met the inclusion criteria and were included in the final meta-analysis.^10^^,^^20^, ^21^, ^22^, ^23^, ^24^ The overall study framework, comparative strategies, and key clinical outcomes are summarized in the Central Illustration.

**Central Illustration:**
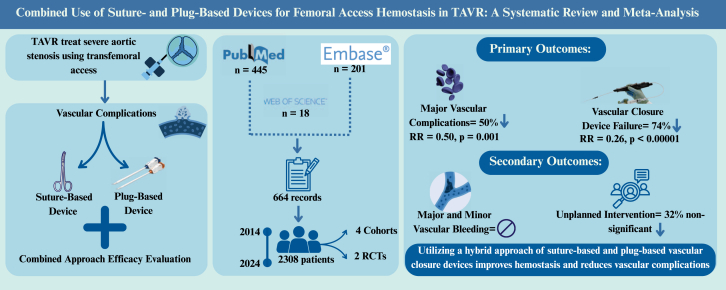
**Comparison of hybrid suture-plus-plug vs dual suture-based vascular closure strategies in transfemoral transcatheter aortic valve replacement (TAVR).** RCT, randomized controlled trial; RR, risk ratio.

### Study characteristics

Our meta-analysis included 4 cohort studies^20^, ^21^, ^22^, ^23^ and 2 RCT^10^^,^^24^ that met the predefined eligibility criteria. The studies were published between 2014 and 2024, with a total of 2308 patients. A summary of the baseline characteristics of the included studies is presented in Tables 1 and 2.^10^^,^^20^, ^21^, ^22^, ^23^, ^24^

**Table 1:** Baseline characteristics of included studies assessing vascular closure strategies in transfemoral TAVR

Reference, year	Study design	Country	Sample size (total)	Women (%)	Follow-up duration	Age, y		BMI, kg/m^2^		CKD, %		CAD, %		Atrial fibrillation, %		LVEF, %		STS score/EuroSCORE	
						Intervention	Control	Intervention	Control	Intervention	Control	Intervention	Control	Intervention	Control	Intervention	Control	Intervention	Control
Kiramijyan et al,^20^ 2016	Retrospective cohort	United States	387	45	In-hospital	82.6 ± 7.6	82.9 ± 8.3	27.6 ± 6.9	27.5 ± 7.2	35.8	42.3	75.6	68.7	44.5	44.6	–	–	–	–
Gmeiner et al,^21^ 2022	Retrospective cohort	Germany	874	45.1	In-hospital	80.8 ± 6.9	80.7 ± 7.7	26.4 ± 4.7	26.6 ± 5.2	9	2	59.7	59.3	39.8	3.0	51.3 ± 9.6	51.1 (± 8.1)	STS score 3.7 ± 2.8	3.8 ± 2.7
Rheude et al,^10^ 2025	RCT	Germany	454	46.9	In-hospital	80.1 ± 6.6	80.3 ± 6.6	26.2 ± 4.9	27.1 ± 4.8	–	–	71.3	77.7	38.7	37.5	–	–	EuroSCORE 4.51 ± 4.28	4.45 ± 4.55
Ko et al,^22^ 2019	Retrospective cohort	China	151	55.6	In-hospital	80.6 ± 7.7	81.8 ± 6.1	24.3 ± 5.4	23.0 ± 3.9	31.0	29.4	37	47.1	17	17.6	65.1 ± 13.1	67.4 ± 11.7	–	–
Cakal et al,^23^ 2022	Retrospective cohort	Turkey	185	44.8	In-hospital	79.07 ± 7	78.91 ± 7.85	25.2 ± 2.82	26.3 ± 3.62	45.7	36	69.6	55.4	21.7	25.2	50.76 ± 11.1	50.97 ± 13.1	EuroSCORE 25.3 (±3.22)	25.99 ± 4.01
Yeh et al,^24^ 2025	RCT	China	257	54.1	In-hospital	81.2 ± 8.2	82.7 ± 7.6	24.2 ± 4.2	24.2 ± 3.8	47	43.8	36.4	33.9	18.9	17.4	63.2 ± 13.4	64.1 ± 13.5	EuroSCORE 5.6 ± 6.7	5.8 ± 9.5

Values are mean ± SD unless noted.

BMI, body mass index; CAD, coronary artery disease; CKD, chronic kidney disease; LVEF, left ventricular ejection fraction; RCT, randomized controlled trial; SD, standard deviation; STS, Society of Thoracic Surgeons; TAVR, transcatheter aortic valve replacement.

**Table 2:** Procedural characteristics and definitions of end points in included studies assessing vascular closure strategies in transfemoral TAVR

Reference, year	Sheath-to-femoral artery ratio		Sheath size, F		Closure device used (n)		Primary end points	Secondary end points
	Intervention	Control	Intervention	Control	Intervention	Control		
Kiramijyan et al,^20^ 2016	1.01 ± 0.17	1.02 ± 0.15	19.32 ± 3.0	20.7 ± 3.3	Dual Perclose + Angio-Seal (208)	Dual Perclose (179)	Primary end points of in-hospital VARC-2 safety outcomes of major and minor vascular complications, VARC-2 life-threatening or disabling bleeding, and VARC-2 major and minor bleeding	Secondary end points included in-hospital serious vascular complications requiring percutaneous or surgical interventions, including vascular perforation or dissection, peripheral ischemia or acute limb ischemia, arteriovenous fistula, pseudoaneurysm, and access-site hematoma
Gmeiner et al,^21^ 2022	–	–	14.3 ± 0.8	14.6 ± 1.0	ProGlide + FemoSeal (437)	ProGlide (437)	Composite of access-related major vascular complications or in-hospital bleeding ≥type 2 according to VARC-3	Overall vascular complications, closure device failure, and bleeding according to the VARC-3 criteria, the need for unplanned surgery or endovascular treatment, and red blood cell transfusion
Rheude et al,^10^ 2025	0.73 ± 0.163	0.75 ± 0.170	–	–	Angio-Seal + ProGlide/ProStyles (230)	Dual ProGlide/ProStyles (224)	Composite of major or minor access site–related vascular complications during index hospitalization according to VARC-3	Time to hemostasis, bleeding type ≥2, and all-cause mortality over 30 d
Ko et al,^22^ 2019	–	–	–	–	Angio-Seal + Dual ProGlide (100)	Dual ProGlide (51)	Vascular access-site complications, defined per VARC-2 criteria, including major (eg, vessel perforation) and minor (eg, small hematoma) complications related to femoral access	Procedure time, contrast medium consumption, fluoroscopy time, arterial injury, need for endovascular intervention
Cakal et al,^23^ 2022	–	–	17.65 ± 1.9	17.02 ± 2.1	Dual Perclose ProGlide + Angio-Seal (46)	Dual Perclose ProGlide (139)	Closure device failure was defined as insufficient or the absence of hemostasis at the arteriotomy site that requires surgical conversion rather than manual compression or adjunctive endovascular intervention	Vascular complications and bleeding were defined using the VARC-2 consensus criteria
Yeh et al,^24^ 2025	0.96 ± 0.02	0.95 ± 0.02	–	–	Angio-Seal + ProGlide (132)	Dual ProGlides (121)	Composite of access site–related major and minor vascular complications during hospitalization for the index procedure, TAVR-related life-threatening bleeding complications within 24 h after the index procedure, and TAVR-related type 2/3 or type 1 bleeding within 24 h after the index procedure according to VARC-3	Deployment of an additional closure device at the femoral access site to achieve hemostasis within 24 h after the index procedure and significant limb ischemia related to arteriotomy closure for TAVR within the 1-y follow-up period

Values are mean ± SD unless noted.

TAVR, transcatheter aortic valve replacement; VARC, Valve Academic Research Consortium.

### Risk of bias assessment

A detailed risk of bias assessment using the RoB 2.0 tool (The Cochrane Collaboration) is given in Supplemental Table S2 and Supplemental Figures S1 and S2.

### Primary outcomes

#### Major vascular complication

The combination of SBD and PBD significantly decreases the risk of major vascular complications (RR, 0.50; 95% CI, 0.33-0.76; *P* = .001; I^2^ = 18%) as compared to suture-based alone (Supplemental Figure S3).

#### VCD failure

Pooled analysis of 6 studies shows that there is a significantly reduced risk of VCD failure by using the combination of SBD and PBD (RR, 0.26; 95% CI, 0.15-0.47; *P* < .00001; I^2^ = 48%) compared to suture-based alone (Supplemental Figure S4).

The leave-one-out sensitivity analysis demonstrated that the pooled effect estimate for VCD failure remained statistically significant and directionally consistent across all iterations, with recalculated RRs ranging from 0.23 to 0.33. Heterogeneity was most notably influenced by the study by Tobias et al,^10^ as its exclusion reduced I^2^ from 48% to 0% (Supplemental Figure S5), whereas exclusion of other studies resulted in I^2^ values ranging from 42% to 58% (Supplemental Table S3). These findings confirm the robustness of the observed reduction in VCD failure with the hybrid suture-plus-plug strategy.

#### Major vascular bleeding

The analysis of major vascular bleeding using a combination of SBD and PBD shows comparable results between the 2 groups (RR, 0.72; 95% CI, 0.39-1.34; I^2^ = 0%, *P* = .30) (Supplemental Figure S6).

### Secondary outcomes

#### Minor vascular complications

Pooled analysis from 6 studies shows that the combination of SBD and PBD significantly reduces the risk of minor vascular complications (RR, 0.70; 95% CI, 0.51-0.96; *P* = .03, I^2^ = 52%) compared to suture-based alone (Supplemental Figure S7).

The leave-one-out sensitivity analysis showed that the direction of effect consistently favored the hybrid strategy across all iterations (RR range, 0.58-0.79). Heterogeneity was primarily driven by the study by Cakal et al^23^ (Supplemental Figure S8), as its exclusion reduced I^2^ from 52% to 0%. Although statistical significance was not maintained after exclusion of several individual studies, the overall trend toward reduced minor vascular complications remained consistent (Supplemental Table S4).

#### Minor vascular bleeding

In the analysis of minor vascular bleeding, the risk was comparable between the 2 groups (RR, 0.86; 95% CI, 0.58-1.28; *P* = .47; I^2^= 0%) (Supplemental Figure S9).

#### Unplanned intervention

In the analysis of unplanned intervention, the risk was comparable between the 2 groups (RR, 0.68; 95% CI, 0.38-1.22; *P* = .19, I^2^ = 46%) (Supplemental Figure S10).

The leave-one-out sensitivity analysis demonstrated consistent directionality favoring the hybrid strategy across all iterations (RR range, 0.54-0.81). Heterogeneity was largely driven by Jonas et al,^21^ as its exclusion reduced I^2^ from 46% to 0% (Supplemental Figure S11). Although the overall pooled estimate remained nonsignificant in most iterations, exclusion of Tobias et al^10^ resulted in a statistically significant reduction in unplanned interventions (RR, 0.54; 95% CI, 0.31-0.95). These findings suggest moderate sensitivity of this end point to individual study effects (Supplemental Table S5).

## Discussion

This systematic review and meta-analysis provides a comprehensive comparison of the efficacy and safety of the suture-plus-plug–based VCD vs the suture-based closure technique for femoral arteriotomy in patients undergoing TF TAVR. Our findings suggest that although both methods carry a comparable risk of major and minor vascular bleeding, the suture-plus-plug–based VCD demonstrates a significantly lower incidence of major and minor vascular complications. Additionally, this hybrid approach is associated with reduced VCD failure rates and a lower need for unplanned interventions, highlighting its potential advantages over the dual suture-based technique.

A recently published meta-analysis by Megantara et al^25^ similarly evaluated hybrid suture/plug-based vs dual suture-based vascular closure strategies following TF TAVR and reported consistent reductions in vascular complications with hybrid approaches. Although our findings align directionally with those prior observations, the present analysis provides several incremental contributions. First, we reported major and minor vascular complications and bleeding outcomes as separated end points, rather than combining them into composite safety measures. Secondly, we performed iterative leave-one-out sensitivity analyses with sequential recalculation of pooled effect estimates and heterogeneity statistics, providing further robustness assessment. Lastly, we adhered strictly to our predefined inclusion criteria, which required explicit specification of the dual SBD. Consequently, studies such as Costa et al^3^ were excluded from ours. Instead, our review incorporates Kiramijyan et al^20^ and Cakal et al,^23^ resulting in a partially distinct study population.

Previous systematic reviews evaluating various VCD provide useful comparisons. A meta-analysis by Sedhom et al^13^ assessing PBD vs SBD for TAVR concluded that PBD were linked to a higher incidence of vascular and bleeding complications. In contrast, our results indicate that integrating a PBD into a suture-based technique lowers the risk of both bleeding and vascular complications compared to the dual suture approach. Notably, our findings also align with those of Sedhom et al^13^ in that they demonstrate a lower incidence of VCD failure with the hybrid technique. Furthermore, although their study reported a higher incidence of unplanned interventions in the PBD group, our analysis suggests that the suture-plus-plug–based approach reduces the need for such interventions. Similarly, a systematic review by Dumpies et al^26^ found no significant difference between SBD and PBD in terms of bleeding events but reported higher VCD failure with PBD—contrasting with our findings.

The advantages of the suture-plus-plug–based approach may stem from its unique mechanism. This combination reduces cinching and strain on the arterial wall, mitigating the risk of complications.^27^ Previous studies^21^^,^^23^ have suggested that this hybrid technique enhances effectiveness and minimizes the likelihood of subsequent peripheral ischemia after TAVR. Several factors contribute to this benefit, including a lower chance of suture tear because of a single suture knob, reduced incidence of incomplete vascular wall apposition, and less interference from vascular calcification, which is a key determinant of VCD failure.^24^ The dual mechanism—mechanical reinforcement via sutures and rapid sealing via the plug—likely underlies the lower complication rates observed. This technique may be particularly advantageous for patients with challenging vascular anatomy, such as small-caliber or heavily calcified femoral arteries, where excessive suturing could increase the risk of stenosis or occlusion.

An ideal VCD would reliably achieve complete hemostasis and arteriotomy closure regardless of arterial wall defect size, patient risk factors, or anticoagulation status. Additionally, it should be easy to use, consistently deploy successfully, and maintain a complication rate equal to or lower than that of manual compression. The device should also facilitate precise targeting of the arteriotomy site to prevent nontarget deployment and avoid risks such as downstream embolization or arterial occlusion. Given that many patients require repeat interventions, an ideal VCD should not induce significant periarterial inflammatory changes that might hinder future access. Other desirable features include bioabsorbable, nonimmunogenic components and cost-effectiveness.^28^ Although the suture-plus-plug–based VCD effectively achieves closure and hemostasis, its deployment is more complex than that of a single-device approach and presents potential device interaction challenges. In contrast, the dual suture–based VCD minimizes inflammatory response and avoids the risks of plug embolization or occlusion but requires a longer time to achieve hemostasis and is technically demanding.

This systematic review offers several strengths. We conducted sensitivity analyses to enhance the robustness of our results, and most included studies were of high methodological quality. In addition, we adhered strictly to our predefined inclusion criteria, ensuring methodological rigor, consistency in study selection, and minimizing the risk of selection bias. However, limitations should be acknowledged. Many included studies were retrospective, introducing potential biases related to patient selection and outcome reporting. Additionally, variations in outcome definitions, such as differing bleeding criteria (VARC-2 vs VARC-3), may have influenced our pooled results. Although sensitivity analyses were performed to address heterogeneity, further standardized trials are necessary to validate these findings. Another limitation is the lack of long-term outcome assessment, preventing us from determining whether the observed benefits of the hybrid approach translate into reduced late complications, such as pseudoaneurysm formation or late reintervention. Future research should focus on long-term follow-up studies and cost-effectiveness analyses to determine whether the initial advantages of the suture-plus-plug approach justify its widespread adoption. Finally, operator experience and procedural techniques play a critical role in VCD success. The dual suture-based approach remains more widely used because of operator familiarity, whereas the hybrid technique is still in an exploratory phase. As more interventionalists gain experience with PBD, outcomes may further improve. Standardized training programs and procedural guidelines should be developed to optimize deployment techniques and minimize complications, ensuring the best possible patient outcomes.

## Conclusion

The combined use of SBD and PBD in TF TAVR is associated with reduced vascular complications and device failure compared to dual suture-based techniques, without an increased risk of bleeding or unplanned intervention. Although these findings support the hybrid approach as a safe and effective strategy, further large-scale randomized trials are warranted to confirm long-term outcomes, assess cost-effectiveness, and establish standardized deployment protocols.

## CRediT authorship contribution statement

**Abdul Moeez:** Conceptualization, Data curation, Methodology, Project administration, Resources, Writing – original draft, Investigation. **Syed Wajihullah Shah:** Conceptualization, Data curation, Formal analysis, Investigation, Methodology, Project administration, Software, Writing – original draft, Writing – review & editing. **Sundus Huma:** Data curation, Formal analysis, Methodology, Software, Validation, Writing – original draft, Writing – review & editing. **Umm E. Salma Shabbar Banatwala:** Data curation, Investigation, Validation, Visualization, Writing – original draft. **Aizaz Ali:** Investigation, Methodology, Supervision, Validation, Visualization, Writing – original draft. **Lintha Zafar Khattak:** Data curation, Investigation, Methodology, Resources, Software, Writing – original draft. **Zaryab Bacha:** Data curation, Formal analysis, Investigation, Methodology, Validation, Visualization, Writing – original draft. **Afra Khan:** Data curation, Investigation, Methodology, Validation, Writing – original draft. **Fazlina Shaid:** Data curation, Methodology, Resources, Software, Validation, Visualization. **Touba Azeem:** Formal analysis, Investigation, Methodology, Validation, Visualization, Writing – original draft. **Sayyam Razzaq:** Data curation, Formal analysis, Investigation, Software, Validation, Writing – original draft. **Malik Waleed Zeb Khan:** Formal analysis, Resources, Supervision, Validation, Writing – original draft, Writing – review & editing. **Jibran Ikram:** Project administration, Resources, Software, Supervision, Validation, Visualization, Writing – review & editing. **Farid Ullah:** Formal analysis, Software, Supervision, Validation, Visualization, Writing – review & editing. **Mohammed Shaban:** Formal analysis, Supervision, Validation, Writing – review & editing, Investigation.

## Footnotes

To access the supplementary material accompanying this article, visit the online version of the *Journal of the Society for Cardiovascular Angiography & Interventions* at 10.1016/j.jscai.2026.105339.
