# Supplementary material for: Hybrid Suture- and Plug-Based Closure Versus Dual Suture Devices in Transfemoral Transcatheter Aortic Valve Replacement: A Systematic Review and Meta-analysis
Source: J Soc Cardiovasc Angiogr Interv. 2026 May 14;5(7):105339. doi: 10.1016/j.jscai.2026.105339 (PMC13400108; doi:10.1016/j.jscai.2026.105339)

**Table S1: Search Strategy of Included Studies**

| **Database** | **Search** | **Items Found** |
| --- | --- | --- |
| Pubmed | (("Aortic Valve Stenosis"[MeSH] OR "Aortic Stenosis"[All Fields] OR "Valve Stenosis"[All Fields]) AND ("Transcatheter Aortic Valve Implantation"[MeSH] OR "TAVR"[All Fields] OR "TAVI"[All Fields] OR "Transcatheter Aortic Valve Replacement"[All Fields]) AND (("Vascular Closure Devices"[MeSH] OR "Closure Devices"[All Fields] OR "Closure Device"[All Fields] OR "Device"[All Fields] OR "Patches"[All Fields] OR "Vascular Closure Device"[All Fields] OR "Vascular Closure Devices"[All Fields] OR "Vascular Closure Patches"[All Fields] OR "Closure Patches"[All Fields] OR "Catheterization Closure Devices"[All Fields] OR "Catheterization"[MeSH] OR "Catheterization Closure"[All Fields]) OR ("Sutures"[MeSH] OR "Suture"[All Fields] OR "Staple"[All Fields] OR "Surgical Staples"[All Fields]) OR ("Peripheral Catheterization"[All Fields] OR "Peripheral Catheterizations"[All Fields] OR "Catheterizations Peripheral Arterial"[All Fields] OR "Peripheral Arterial Catheterization"[All Fields] OR "Peripheral"[All Fields] OR "Catheterizations"[All Fields])) AND ("Postoperative Hemorrhage"[MeSH] OR "Postoperative"[All Fields] OR "Postoperative Hemorrhages"[All Fields] OR "Hemorrhage"[All Fields] OR "Blood Loss"[All Fields] OR "Postoperative Blood"[All Fields] OR "Postoperative Blood Loss"[All Fields]) AND ("Treatment Outcome"[MeSH] OR "Outcome"[All Fields])) | 445 |
| Embase | ('aortic valve stenosis'/exp OR 'aortic stenosis':ab,kw OR 'valve stenosis':ab,kw) AND ('transcatheter aortic valve implantation'/exp OR 'TAVR':ab,kw OR 'TAVI':ab,kw OR 'transcatheter aortic valve replacement':ab,kw) AND (('vascular closure device'/exp OR 'closure devices':ab,kw OR 'closure device':ab,kw OR 'device':ab,kw OR 'patches':ab,kw OR 'vascular closure patch':ab,kw OR 'vascular closure patches':ab,kw OR 'closure patches':ab,kw OR 'catheterization closure device':ab,kw OR 'catheterization'/exp OR 'catheterization closure':ab,kw) OR ('suture'/exp OR 'sutures':ab,kw OR 'staple':ab,kw OR 'surgical staples':ab,kw) OR ('peripheral catheterization':ab,kw OR 'peripheral catheterizations':ab,kw OR 'catheterizations peripheral arterial':ab,kw OR 'peripheral arterial catheterization':ab,kw OR 'peripheral':ab,kw OR 'catheterizations':ab,kw)) AND ('postoperative hemorrhage'/exp OR 'postoperative':ab,kw OR 'postoperative hemorrhages':ab,kw OR 'hemorrhage':ab,kw OR 'blood loss':ab,kw OR 'postoperative blood':ab,kw OR 'postoperative blood loss':ab,kw) AND ('treatment outcome'/exp OR 'outcome':ab,kw) | 201 |
| Web of science | TS=("Aortic Valve Stenosis" OR "Aortic Stenosis" OR "Valve Stenosis") AND TS=("Transcatheter Aortic Valve Implantation" OR "TAVR" OR "TAVI" OR "Transcatheter Aortic Valve Replacement") AND TS=(("Vascular Closure Devices" OR "Closure Devices" OR "Closure Device" OR "Device" OR "Patches" OR "Vascular Closure Device" OR "Vascular Closure Patches" OR "Closure Patches" OR "Catheterization Closure Devices" OR "Catheterization Closure" OR "Catheterization") OR ("Sutures" OR "Suture" OR "Staple" OR "Surgical Staples") OR ("Peripheral Catheterization" OR "Peripheral Catheterizations" OR "Catheterizations Peripheral Arterial" OR "Peripheral Arterial Catheterization" OR "Peripheral" OR "Catheterizations")) AND TS=("Postoperative Hemorrhage" OR "Postoperative" OR "Postoperative Hemorrhages" OR "Hemorrhage" OR "Blood Loss" OR "Postoperative Blood" OR "Postoperative Blood Loss") AND TS=("Treatment Outcome" OR "Outcome") | 18 |

| Study ID | Selection | | | | Comparability | | Outcome | | | Total | Study Quality |
| --- | --- | --- | --- | --- | --- | --- | --- | --- | --- | --- | --- |
|  | S1 | S2 | S3 | S4 | C | | O1 | O2 | O3 |  |  |
| Cakal et al 2022 | * | * | * | - | * | * | * | * | * | 8* | Good  quality |
| Sarkis et al 2016 | * | * | * | - | * | * | * | * | * | 8* | Good  quality |
| Jonas et al 2022 | * | * | * | - | * | * | * | * | * | 8* | Good  quality |
| Ko et al 2019 | * | * | * | - | - | - | * | * | * | 6* | Poor  quality |

**Table S2: Assessment of Study Quality Using the Newcastle-Ottawa Scale (NOS)**

**Supplementary Table S3. Leave-One-Out Sensitivity Analysis for Vascular Closure Device Failure**

| **Study Excluded** | **Risk Ratio** | **95% CI** | **P-value** | **I² (%)** |
| --- | --- | --- | --- | --- |
| \| None (All studies included) \|  \|  \|  \|  \| \| --- \| --- \| --- \| --- \| --- \| | 0.26 | 0.15-0.47 | <0.00001 | 48 |
| \| Cakal et al. \|  \|  \|  \|  \| \| --- \| --- \| --- \| --- \| --- \| | 0.26 | 0.14-0.50 | <0.0001 | 58 |
| \| Jonas et al. \|  \|  \|  \|  \| \| --- \| --- \| --- \| --- \| --- \| | 0.26 | 0.13-0.51 | 0.0001 | 57 |
| \| Ko et al. \|  \|  \|  \|  \| \| --- \| --- \| --- \| --- \| --- \| | 0.23 | 0.14-0.39 | <0.00001 | 42 |
| \| Sarkis et al. \|  \|  \|  \|  \| \| --- \| --- \| --- \| --- \| --- \| | 0.26 | 0.13-0.52 | 0.0002 | 56 |
| \| Tobias et al. \| \| --- \| | 0.33 | 0.21-0.51 | <0.00001 | 0 |
| \| Yeh et al. \| \| --- \| | 0.27 | 0.12-0.59 | 0.001 | 52 |

**Supplementary Table S4. Leave-One-Out Sensitivity Analysis for Minor Vascular Complications**

| **Study Excluded** | **Risk Ratio** | **95% CI** | **P-value** | **I² (%)** |
| --- | --- | --- | --- | --- |
| \| None (All studies included) \|  \|  \|  \|  \| \| --- \| --- \| --- \| --- \| --- \| | 0.70 | [0.51, 0.96] | 0.03 | 52 |
| \| Cakal et al. \|  \|  \|  \|  \| \| --- \| --- \| --- \| --- \| --- \| | 0.58 | [0.48, 0.71] | <0.00001 | 0 |
| \| Jonas et al. \|  \|  \|  \|  \| \| --- \| --- \| --- \| --- \| --- \| | 0.71 | [0.48, 1.04] | 0.08 | 61 |
| \| Ko et al. \|  \|  \|  \|  \| \| --- \| --- \| --- \| --- \| --- \| | 0.72 | [0.50, 1.03] | 0.07 | 62 |
| \| Sarkis et al. \|  \|  \|  \|  \| \| --- \| --- \| --- \| --- \| --- \| | 0.70 | [0.47, 1.04] | 0.08 | 58 |
| \| Tobias et al. \| \| --- \| | 0.79 | [0.57, 1.09] | 0.14 | 24 |
| \| Yeh et al. \| \| --- \| | 0.73 | [0.49, 1.08] | 0.12 | 62 |

**Supplementary Table S5. Leave-One-Out Sensitivity Analysis for Unplanned Interventions**

| **Study Excluded** | **Risk Ratio** | **95% CI** | **P-value** | **I² (%)** |
| --- | --- | --- | --- | --- |
| \| None (All studies included) \|  \|  \|  \|  \| \| --- \| --- \| --- \| --- \| --- \| | 0.68 | [0.38, 1.22] | 0.19 | 46 |
| \| Cakal et al. \|  \|  \|  \|  \| \| --- \| --- \| --- \| --- \| --- \| | 0.64 | [0.32, 1.27] | 0.20 | 58 |
| \| Jonas et al. \|  \|  \|  \|  \| \| --- \| --- \| --- \| --- \| --- \| | 0.81 | [0.55, 1.18] | 0.27 | 0 |
| \| Ko et al. \|  \|  \|  \|  \| \| --- \| --- \| --- \| --- \| --- \| | 0.68 | [0.35, 1.32] | 0.25 | 59 |
| \| Tobias et al. \| \| --- \| | 0.54 | [0.31, 0.95] | 0.03 | 22 |
| \| Yeh et al. \| \| --- \| | 0.67 | [0.25, 1.80] | 0.42 | 60 |

**Figure S1: Risk of Bias Summary for Individual Studies**


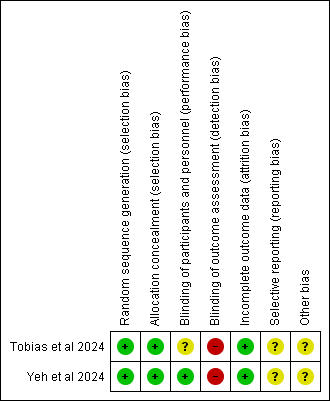


**Figure S2: Risk of Bias Graph for Included Studies**


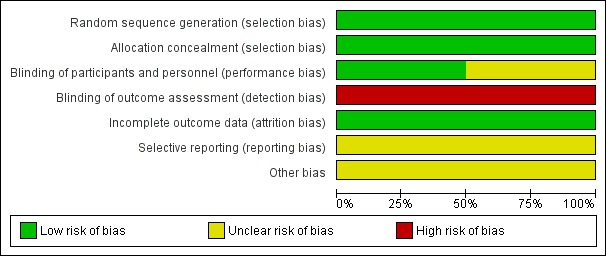


**Figure S3: Forest plot of major vascular complications.**

**
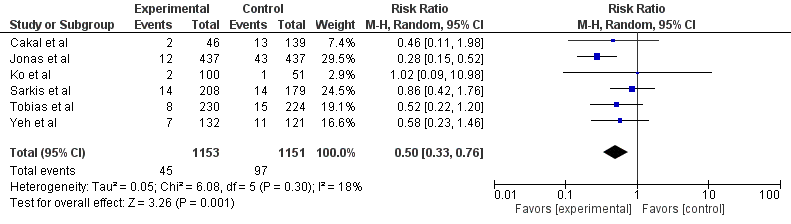
**

**Figure S4: Forest plot of vascular closure device failure.**

**
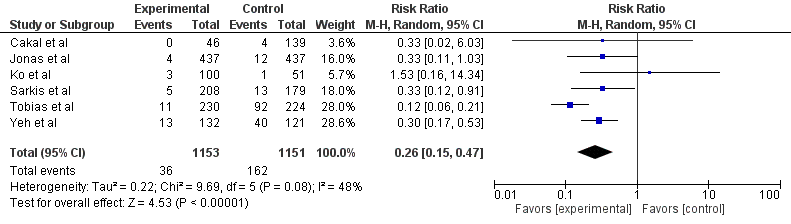
**

**Figure S5: Sensitivity analysis of vascular closure device (VCD) failure.**


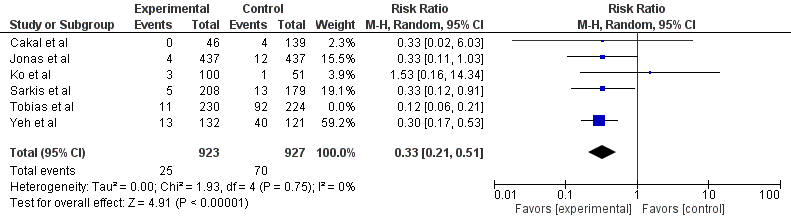


**Figure S6: Forest plot of major vascular bleeding.**

**
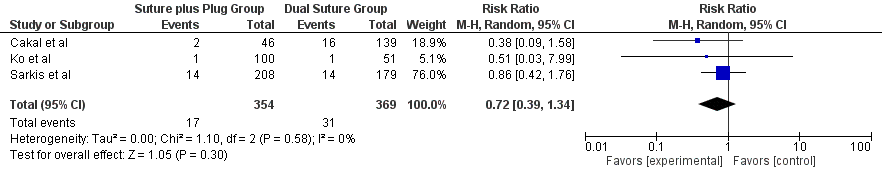
**

**Figure S7: Forest plot of minor vascular complications**

**
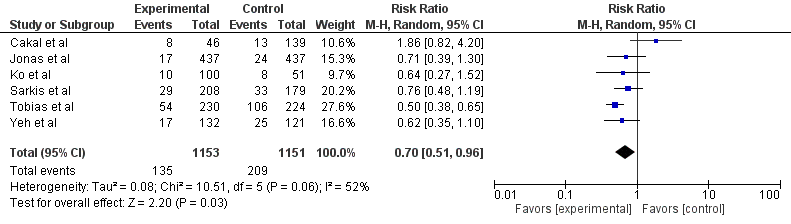
**

**Figure S8: Sensitivity analysis of minor vascular complications.**


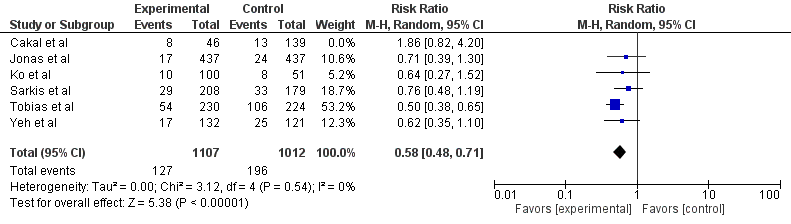


**Figure S9: Forest plot of minor vascular bleeding.**

**
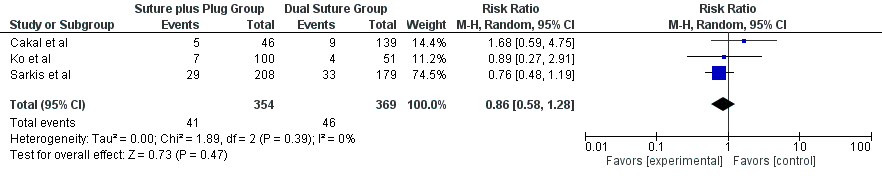
**

**Figure S10: Forest plot of unplanned interventions.**

**
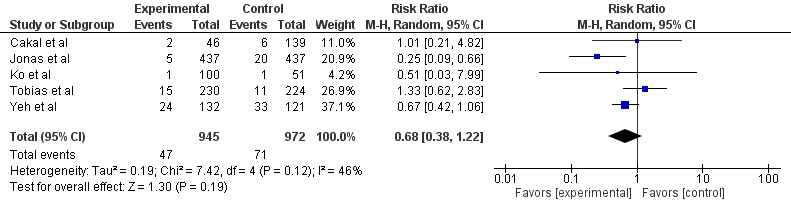
**

**Figure S11: Sensitivity analysis of unplanned surgical interventions.**


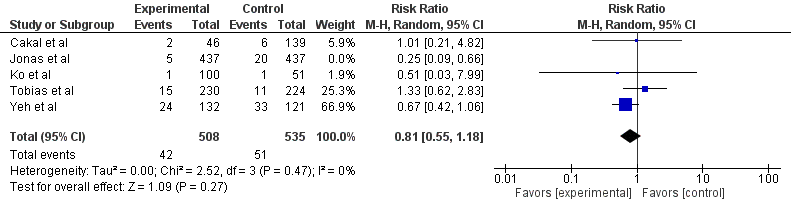

Supplement: Supplementary Data [file mmc1.docx]
